# Characterization of Thermoplastic Polyurethane (TPU) and Ag-Carbon Black TPU Nanocomposite for Potential Application in Additive Manufacturing

**DOI:** 10.3390/polym9010006

**Published:** 2016-12-29

**Authors:** Steven T. Patton, Chenggang Chen, Jianjun Hu, Lawrence Grazulis, Amanda M. Schrand, Ajit K. Roy

**Affiliations:** 1 Nonstructural Materials Division, University of Dayton Research Institute, Dayton, OH 45469-0050, USA; chenggang.chen.ctr@us.af.mil (C.C.); jianjun.hu.1.ctr@us.af.mil (J.H.); general_info@udri.udayton.edu (L.G.); 2 Munitions Directorate, Air Force Research Laboratory, Eglin Air Force Base, Valparaiso, FL 45433-7750, USA; amanda.schrand.2@us.af.mil; 3 Materials & Manufacturing Directorate, Air Force Research Laboratory, Wright-Patterson Air Force Base, Dayton, OH 45433-7750, USA; ajit.roy@us.af.mil

**Keywords:** thermoplastic polyurethane, nanocomposite, compression, electromechanical response, viscoelasticity, adhesion, chain relaxation/ordering, additive manufacturing

## Abstract

Electromechanical, adhesion, and viscoelastic properties of polymers and polymer nanocomposites (PNCs) are of interest for additive manufacturing (AM) and flexible electronics. Development/optimization of inks for AM is complex, expensive, and substrate/interface dependent. This study investigates properties of free standing films of a thermoplastic polyurethane (TPU) polymer and an Ag–carbon black (Ag-CB) TPU PNC in a lightly loaded low strain compression contact as a rough measure of their suitability for AM. The TPU exhibited high hysteresis and a large viscoelastic response, and sufficient dwell time was needed for polymer chain relaxation and measurable adhesion. A new discovery is that large enough contact area is needed to allow longer time constant polymer ordering in the contact that led to higher adhesion and better performance/reliability. This has previously unknown implications for interface size relative to polymer chain length in AM design. The standard linear model was found to be a good fit for the viscoelastic behavior of the TPU. The PNC exhibited no adhesion (new result), low electrical resistance, and relatively small viscoelastic response. This implies potential for AM electrical trace as well as switch applications.

## 1. Introduction

Additive manufacturing (AM) of flexible electronics offers promise for fabrication of large area, low cost, and lightweight microelectronic circuits and durable devices on flexible conformal polymer substrates at mass production rates [1,2]. Additional capabilities created by AM include complex new circuit designs and printing on irregular/flexible surfaces to optimize limited space. Flexible electronics applications include wearable and conformal systems, which tie in to human effectiveness, robotics, prosthetics, healthcare, and touch sensors [3]. Reliability is a concern in many applications due to stretching, compression, bending, and harsh aerospace environments (e.g., space, humidity, thermal cycling, mechanical shock, etc.). Interface adhesion between printed materials (e.g., metallic or polymer nanocomposite (PNC) traces/interconnects, nanoparticles, dielectrics/insulators, semiconductors, etc.), substrates (typically polymers such as polyethylene terephthalate (PET), polycarbonate, or polyimide (PI)), and contacts (Au, Ag, Cu, Al, etc.) is important for print quality and reliability. This study aims to provide needed fundamental studies that investigate electromechanical, viscoelastic, and adhesion properties of potentially relevant materials/pairs to support AM ink development efforts. 

Nanocomposite elastomers are being developed for printed electrically conductive traces and die attach/solder replacements for flexible electronics boards [4,5,6]. Conductive tracks are regularly printed on plastic sheets, such as PET, polycarbonate, or PI films [2,7]. Low electrical conductivity of printed conductive metal contacts and traces is one of the primary problems that needs to be solved, and to improve the conductivities of printed tracks, efforts have been made to improve nanostructures of conductive material, ink formulations, and printing procedures [7]. There is a need for a better understanding of conduction mechanisms within the trace materials and this is an objective of the present study.

Inkjet printing is a common AM technique and is a promising alternative production tool for manufacturing conductive components in electronic devices [1,7]. There are many benefits associated with inkjet printing (compared to other deposition methods) including low-cost, its additive nature, efficient handling of expensive materials, one-step processing, cheap and compact equipment, and applicability to various substrates [7,8]. The availability of metal printing ink and its performance is key to the success of printing technology in the electronics industry [9]. Commonly used inks for the inkjet printing of conductive components are conducting polymers and nanoparticle (NP) solutions with dispersants all of which have associated drawbacks [7]. Gold, copper, and silver nanoparticles are being considered for printed devices [2]. Nanomaterials are currently not used extensively in AM processes due to their cost, their environmental stability, their tendency to agglomerate and settle, and the need to “tune” the AM processing conditions [10]. Development and optimization of a successful ink for ink jet printing is complex and expensive and involves selection of the proper metal particles (<5 nm size for depressed melting temperature and low annealing temperature), solvent, surfactant, and adhesion promoter (unique to each substrate) [9]. 

Poor electrical performance, mechanical strength, and adhesion (e.g., to epoxy molding compounds) limits interest in printing technology for the semiconductor industry [9]. Simple and inexpensive pre-screening, property evaluation, and development of AM electronic materials at the free-standing film level seems prudent so that expensive and complicated ink development is performed for only the most promising materials. However, it should be pointed out that the experimental result of a free standing film may not be representative of the properties of an AM film. Electromechanical, viscoelastic, and adhesion are some of the most important properties that should be evaluated before cumbersome and expensive ink development is undertaken. This approach was taken in a previous study where mechanical properties (e.g., hardness and elastic modulus) of printable polyurethane (PU) was evaluated using cast specimens [11]. Extension of this approach to include all of electromechanical, viscoelastic, and adhesion properties of film specimens is a goal of this study. An additional motivation for free-film level studies is that a material property(s) can be linked to good printing performance giving insight into associated mechanisms. Free-film properties can also be compared with printed material properties as a measure of print quality. Since the surface characteristics and micro/nanostructure of the films prepared by casting and AM are likely to differ, the applicability of the results to AM needs to be put into context. The casted films are more representative of bulk properties and may be a good indicator for applicability to AM, especially in the context of their ultimate performance. However, casted and AM films are fabricated in different ways and their properties may differ. An example would be different internal stress that could affect properties such as the influence of moisture/water.

One challenge to the attainment of satisfactory device performance is the adhesion and mechanical strength of the printed tracks on flexible substrates [2]. Most commonly used conductive inks contain electronically non-conductive binders to help the adhesion and mechanical stability of printed patterns on substrates [2]. Thus, more investigation on improving the adhesion of metal tracks on plastic substrates while maintaining high conductivity is still needed [2]. There is interest in the underlying mechanisms of adhesion between polymers and interface materials, such as polymer-to-polymer, polymer-to-metal, polymer-to-ceramic, or polymer-to-inks, -coatings, and -adhesives [6]. 

PU elastomers are widely-used because of their excellent mechanical properties (e.g., tear and abrasion resistance, and flex-fatigue life), biocompatibility, adherence to substrates, and other desirable and tailorable characteristics [11,12,13,14]. PU elastomers are also used as shock isolators, particularly in extreme applications with large deformation and high mechanical impact [14]. There is considerable interest in using PUs in AM, for instance, reactive inkjet printing was used to generate gradient material made from PU [11]. The mechanical properties of PU elastomers were found to be highly dependent on temperature and humidity with significant softening with increase in temperature and humidity levels [13]. The mechanical and viscoelastic properties of PU depend on the microphase separation of hard and soft domains that comprise the polymer [15]. Creep failure mechanisms are important for plastics and include cracking associated with creep rupture as well as dimensional changes to parts making them unusable [16].

Since adhesion of layers in AM is essential for mechanical integrity and device reliability, an understanding of the underlying adhesion mechanisms is essential. Dwell time and contact force are two important factors to consider as to their effect on adhesion. The influence of dwell time on the adhesion strength for an uncrosslinked styrene-butadiene (SBR) rubber in contact with fused silica was investigated, and a slight increase in the work of debonding was shown with increased dwell time due to the relatively slow diffusion of trapped air away from the interfaces as well as the relaxation of SBR polymer chains at the interface, which form a better contact with the surface of the glass the longer they are left in contact [17]. The trapped air pockets served as crack nucleation sites at short contact times and led to lower adhesion [17]. 

The aim of this study was to investigate the electromechanical, viscoelastic, and adhesion properties of free standing thermoplastic polyurethane (TPU) and carbon black-decorated with silver (Ag-CB) TPU PNC films in a lightly loaded and low strain compression contact between Au films. This work also serves as an example of a way to prescreen and evaluate material properties to potentially determine/develop suitability for AM before expensive and cumbersome ink development is undertaken. The methodology can be used to evaluate various material pairs of interest for AM. For the neat TPU, force displacement curves show high adhesion force as well as high hysteresis suggesting potential for good interface reliability and suitability as a viscoelastic damping material for shock isolator applications, respectively. Adhesion for the neat TPU generally increases with both initial contact force and dwell time, but is absent at a short dwell time of 500 ms due to the time constants (τ) for short chain segment rearrangement/relaxation (short τ) and chain diffusion/rearrangement/ordering (long τ) across the interface being greater than 500 ms. The ordering energy minimization mechanism became operative at high contact force/area and high dwell time due to the need for sufficient contact area (since chains are extended with ordering) and time to form the ordered structures. This could have implications in AM structure design as interface size must be large enough (compared to polymer chain length) to ensure adequate adhesion. 

For the PNC, no adhesion and minimal hysteresis were observed due to small real area of contact (RAC) and load support through spanning conductive paths (as opposed to the polymer), respectively. Low and constant resistance (0.4 Ω) was measured at higher strain (from 2% to 3%), which suggests a metallic conductive path(s), and was associated with a mild increase in stiffness due to larger radii of curvature of phase separated Ag structures in the composite. Both the TPU and PNC show good promise for AM and are candidates for further development as ink materials.

## 2. Experimental Section

**Figure 1:**
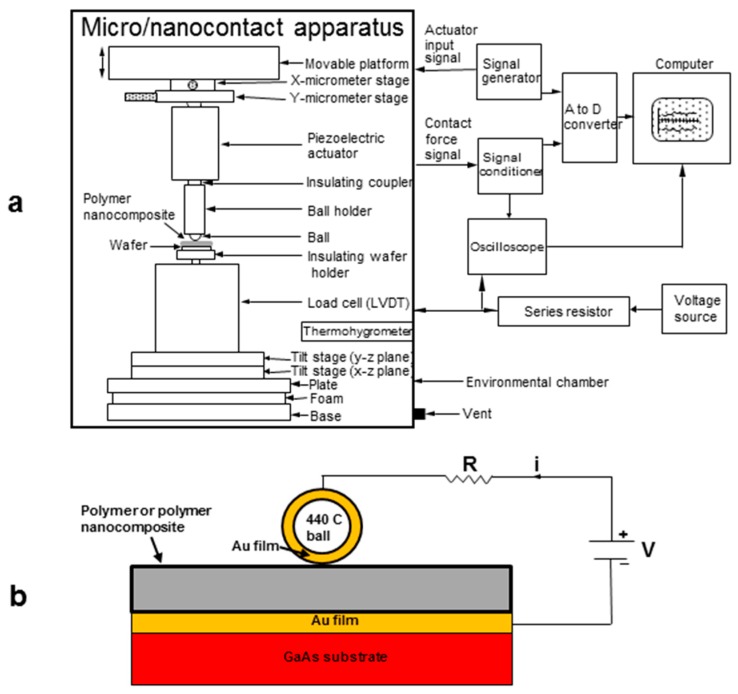
(**a**) Schematic of the micro/nanocontact apparatus and associated instrumentation used in polymer and PNC compression experiments; (**b**) Close up view of the ball-polymer or PNC-wafer contact along with the electric circuit used in this study.

A micro/nanocontact apparatus was used for this study and is shown schematically in Figure 1a. The apparatus was described in detail in earlier studies that investigated the electromechanical behavior of carbon fibers, carbon nanotube (CNT)-coated carbon fibers (fuzzy fibers), and multiwalled carbon nanotube (MWCNT) TPU PNCs (for tactile sensors) [18,19]. This type of test was selected due to its ability to measure small compressive force and displacement along with the ability for electric current flow and electrical resistance measurement. This makes the test suitable for multiphysics studies. Although this type of indentation method is not new for characterizing adhesion and viscoelasticity, the mesoscale capabilities of the apparatus used here can provide data not possible with macroscale and nanoscale test apparatus. Briefly, a ball-on-flat electrode configuration is used with Au-coated GaAs wafers and Au-coated 1.6 mm diameter Grade 100 440C stainless steel balls. The polymer or PNC was sandwiched between the electrodes in compression experiments as shown in Figure 1b. Peak forces used were generally less than 20 mN and peak relative displacements (*RD*s) less than 10 μm. A thermohygrometer was used to monitor the temperature and relative humidity (*RH*) inside the chamber. The temperature and RH inside the chamber during experiments were maintained at 22 ± 1 °C and 1 ± 0.1%, respectively. 

Figure 1b shows a close up view of the ball-polymer-wafer or ball-PNC-wafer contact along with the electrical circuit used for electrical resistance measurements. Compression experiments on the neat polymer or PNC sandwiched between Au electrodes were conducted using a piezoelectric actuator for ball displacement. Either a triangular or square wave excitation voltage was used to drive the actuator. Different frequencies were used to vary the strain rate. Controlled displacement of the ball electrode initiated polymer or PNC compression against the flat electrode, and the resulting compression force and displacement of the lower electrode were measured with the high sensitivity load/displacement cell shown in Figure 1a. A constant open circuit voltage of 10 V was used along with a fixed resistor of 1 kΩ assembled in series with gold plated electrodes to limit the current to less than 10 mA. Voltage drop across the series resistor was monitored with an oscilloscope and stored in a computer. Simple circuit analysis was used to calculate the electrical resistance of the sandwiched PNC. The maximum measurable electrical resistance with the circuit and instrumentation is 10^9^ Ω. The above procedure was found to be a reliable way to conduct the experiments.

The fabrication of the Ag-CB TPU PNC film is described in the following. Estane^®^ 5719 TPU (Lubrizol, Cleveland, OH, USA) was used in this study, and is a polyester-type TPU. The average molecular weight is 122,000. This material was selected due to its relatively high tensile strength, high tear strength, low temperature flexibility, excellent dynamic fatigue properties, high adhesion to various substrates such as PET, and its potential for strain resilient electronic interconnect materials [14,19,20]. Estane 5719 TPU also has higher hardness and better scratch resistance than Morthane TPU, making it attractive for printed electronics. The Ag-CB was produced by Materials Research Institute (MRI), Limited Liability Company (LLC), Dayton, OH, USA. The silver NP diameter is 30–40 nm, and the CB diameter is 15–20 nm. The Ag-CB was mixed with TPU in the presence of a solvent of dibasic ester (DBE) via high shear mixing, followed by the three-roll-milling procedure, performed by MRI, LLC. Then, the Ag-CB/TPU/DBE mixture was further processed via Thinky mixing. The mixture was cast in a silicone mold and heated in an autoclave as follows: 75 to 250 °F at a ramp rate of 2 °F/min, hold at 250 °F for 3 h, then cool down to 75 °F. The vacuum was kept at −27 inch Hg and the pressure was kept at +120 psi. The vacuum and pressure were released after the parts were cooled. Samples were prepared with 88 wt % Ag-CB loading (81 wt % Ag and 7 wt % CB). PNC sample sheets were 220 μm thick and were cut into 5 mm × 5 mm rectangles for testing. Pure TPU samples were 280 μm thick and also cut into 5 mm × 5 mm rectangles for testing.

**Figure 2:**
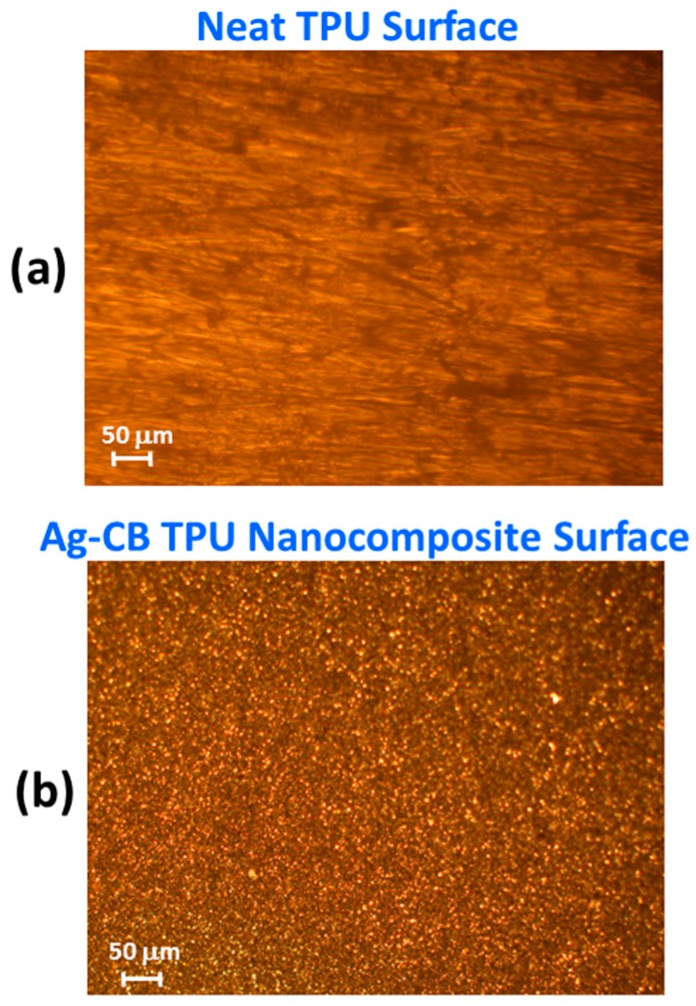
Optical microscope images of: (**a**) the neat TPU surface; and (**b**) the Ag-CB TPU PNC. The TPU is relatively featureless compared to the grainier and rougher appearing PNC with many asperities.

Optical micrographs of the neat TPU and Ag-CB TPU PNC are shown in Figure 2. The TPU surface is relatively featureless compared to the grainier and rougher looking PNC surface that has many asperities. The Ag-CB TPU PNC sample surface was further examined for surface topography and surface roughness using atomic force microscopy (AFM). The equipment used for this experiment was a commercial AFM, Park Scientific Instruments NX-10 system using a NSC15/Al BS cantilever (Innovative Solutions Bulgaria Limited, Sofia, Bulgaria), with a tip radius of less than 7 nm which oscillates with the resonance frequency of the cantilever ω_res_ = ~325 kHz. Non-contact mode was used for all imaging with a slow scan speed to accommodate the maximum deviation in the sample topography.

**Figure 3:**
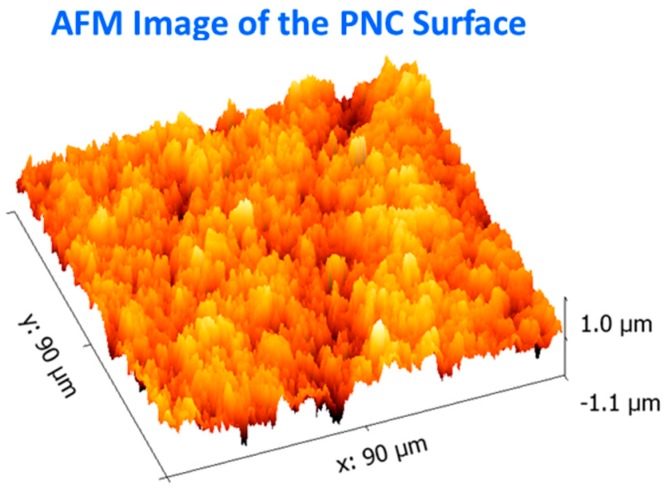
AFM image of the Ag-CB TPU PNC surface showing surface asperities.

An AFM surface image of the PNC is shown in Figure 3. Many asperities are seen on the PNC surface. A rough surface contact and associated contact mechanics are applicable when the PNC surface contacts the gold electrodes. The implications of contact mechanics for PNCs during compression experiments were discussed thoroughly in an earlier study [19]. The outer surface of the PNC is important for making electrical contact for electrical interconnect applications. In the case of the TPU, its low modulus and high compliance allows it to readily deform into a conformal contact with the high modulus Au electrodes with high RAC [21].

## 3. Results and Discussion

**Figure 4:**
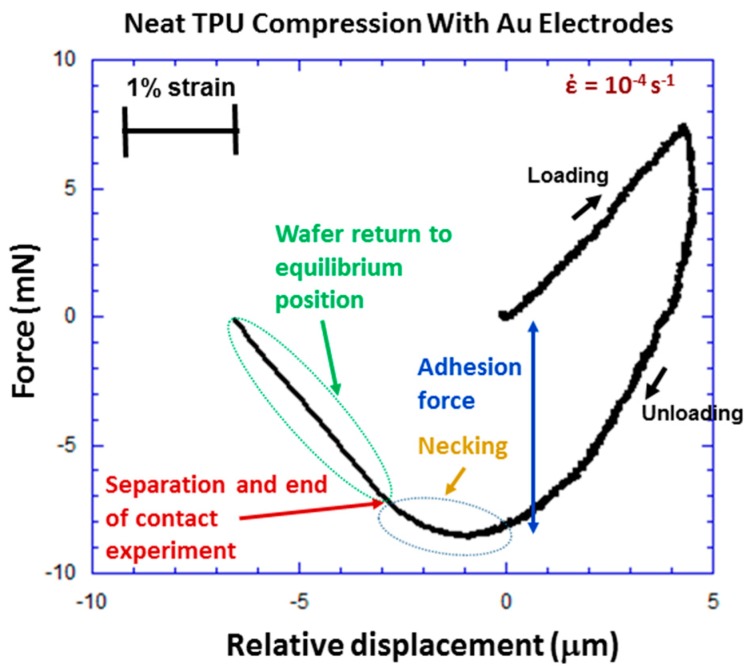
Contact force versus RD for the neat TPU polymer during a localized compression experiment. Hysteresis, adhesion, and necking are observed in the experiment.

The result of a compression experiment for the neat TPU polymer is shown in Figure 4. The experiment uses a triangular wave voltage excitation to drive the ball actuator. Force is plotted as a function of *RD*. *RD* is the compressive displacement or penetration imposed on the TPU specimen at the ball apex. The relationship between *RD* and strain (ε) on the PNC specimen at the ball apex is

ε = *RD*/*L*, (1)

where *L* is the sample thickness. The strain scale bar in Figure 4 is based on the TPU sample thickness of 280 μm. Contact area is important for adhesion characterization and the geometric relationship between contact area (*A*) and RD is

*A* = 2.5 × 10^−3^*RD*. (2)

The strain rate in Figure 4 is 10^−4^·s^−1^ and corresponds to a quasistatic experiment.

The loading curve for the TPU in Figure 4 is linear up to the maximum RD of about 4 μm. However, upon unloading, a large amount of hysteresis is observed, which is consistent with the results of another study [22]. The observed large hysteresis in Figure 4 suggests that the TPU would be a good candidate for shock isolator applications [14]. Internal friction due to relative sliding of the polymer chains or viscoelastic dissipation is a contributor to energy loss and hysteresis, and is related to TPU resilience [15,22,23]. The viscoelastic dissipation behavior of the TPU originates from several energy dissipation sources including: disentanglement of soft segment chains, pull out of soft segment chains from hard domains, plastic slip in hard domains, the breakage of hydrogen bonds in the hard domains, frictional interactions and break up of two hard domains, and interactions between soft and hard domains [15,22]. The extent of the strain and mode of deformation determines which mechanisms are most important. During compression, free volume of the polymer is reduced and is associated with increased order and viscoelastic deformation. This is expected to be important in this paper where compression at low strain is the mode of deformation. 

An adhesion force of about 8.5 mN is observed in Figure 4. The presence of this large adhesion force suggests that adhesion hysteresis may contribute to the hysteresis in Figure 4. The energy loss mechanism in adhesion hysteresis is a result of nonequilibrium processes occurring at the interface [24]. Inelastic deformation, diffusion, disentanglement, rearrangement, relaxation, and orientation (ordering) of polymer chains at the interface to form a better contact with the counter surface are thought to be a contributor to high adhesion and adhesion hysteresis [17,24]. Necking is observed during unloading from RD = 0 to −3 μm. The contact breaks at RD = −3 μm which is the end of the contact experiment, after which the wafer returns to its equilibrium position. The presence of such strong necking is due to the high adhesion between the Au film and the polymer. As the polymer necks out, the stiffness *k* of the neck decreases according to

*k* = *EA*/*L* (3)

due to a decrease in *A* and increase in *L*, where *E* is the elastic modulus, *A* is the cross sectional area, and *L* is the length. This decrease in neck stiffness is seen in the unloading curve from *RD* = 0 to −3 μm.

Since significant adhesion is evident in Figure 4, the mechanism of adhesion between the TPU and Au film is of considerable interest. High adhesion could positively impact the performance (better bonding between nanofillers and the polymer matrix) and reliability (better adhesion between layers, substrate, etc.) of printed material. Thus, an adhesion experiment was needed such that the initial contact force (contact area) and dwell time could be controlled independently and varied to study their effect. For challenging and difficult adhesion measurements, it is also important that the approach and retraction velocity be the same from experiment-to-experiment to ensure consistency and since the measured adhesion force is known to vary with a number of experimental parameters including separation velocity [21]. A square wave ball excitation was chosen where the frequency of the drive voltage was used to determine the dwell time. The initial contact force was determined by the initial separation between the ball and flat sample, with the drive voltage amplitude being held constant at 10 V (8 μm actuator displacement). The strain rate was 1 s^−1^ during loading and unloading in square wave experiments. One strength of the experimental system in Figure 1 is its mesoscale character, bridging the gap between AFM and macroscale measurement systems. Thus, this study is able to apply and measure contact/adhesion forces on the mN scale or below providing excellent sensitivity, while establishing a large enough contact area to give reliable data. 

**Figure 5:**
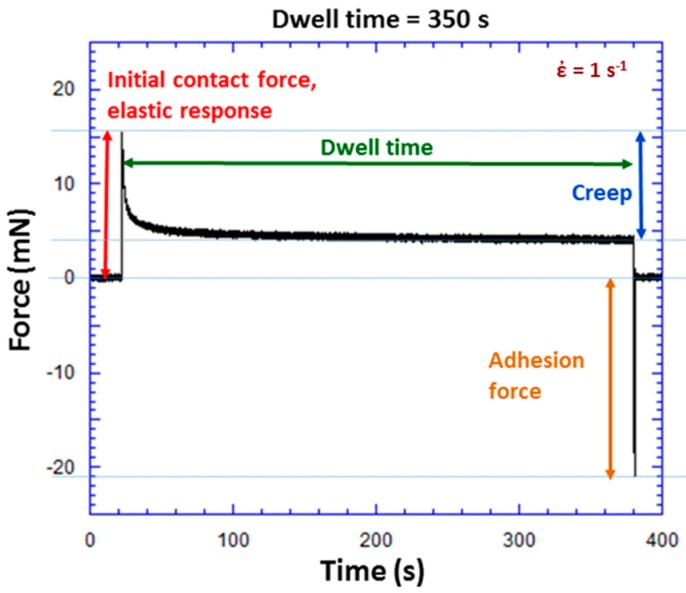
Force versus time during a TPU experiment with a square wave ball excitation. This type of experiment was used to measure adhesion force at various initial contact forces and dwell times. For this particular experiment at a dwell time of 350 s, initial contact force was 15.8 mN and adhesion force 22.1 mN.

Figure 5 shows an example of an experiment to determine adhesion force with a dwell time of 350 s. The initial contact force, which is an elastic response to the forced contact between the ball and TPU, is 15.8 mN for this particular experiment. During the dwell time period, ball position is held constant. Force decreases in Figure 5 from 15.8 mN at *t* = 25 s to 4.1 mN at *t* = 375 s. This force relaxation is associated with viscoelastic creep. With ball position being held constant, wafer displacement upwards is the only explanation for the observed force relaxation. The upward wafer displacement corresponds with an increase in the contact area. An adhesion force of 22.1 mN is measured upon separation of the ball and wafer as shown in Figure 5. This is the method used to measure adhesion force over a range of initial contact forces and dwell times.

**Figure 6:**
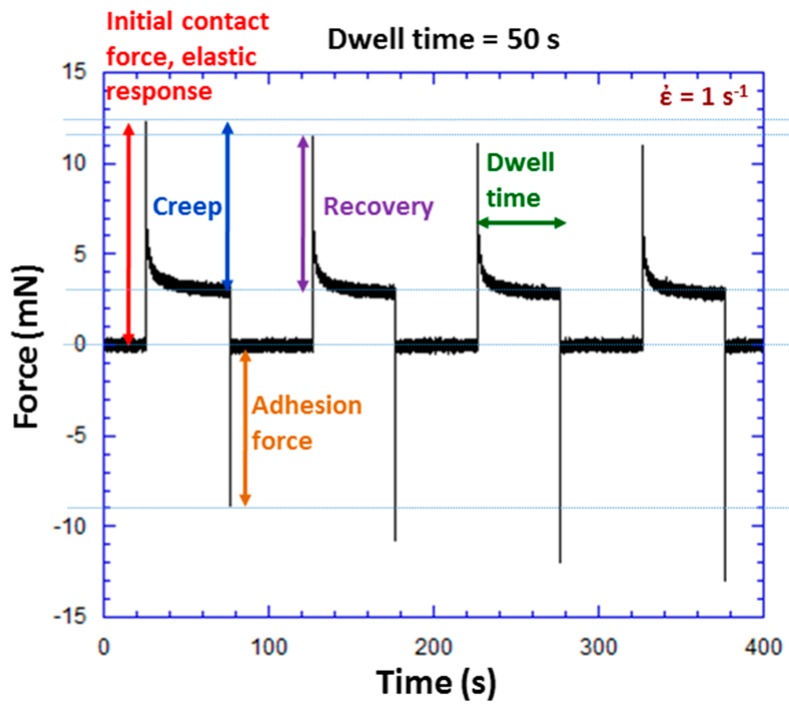
Force versus time during a TPU experiment with a square wave ball excitation. For this particular experiment at a dwell time of 50 s, initial contact force was 12.3 mN and adhesion force 9.1 mN.

Figure 6 shows another example of an experiment to determine adhesion force, but with a dwell time of 50 s. Four consecutive contact events are shown in Figure 6. The initial contact force is 12.3 mN and adhesion force 9.1 mN for the first contact event. Creep (force relaxation), recovery of viscoelastic deformation, and adhesion are all illustrated in Figure 6. Recovery of viscoelastic deformation is illustrated by the force difference from the end of one contact event to the beginning of the next contact event. Viscoelastic recovery is not complete after being out of contact for 50 s between contact events. The residual deformation increases with each contact cycle and is evident in the decreasing elastic response with increasing contact cycles. It is interesting to note that the adhesion force increases as the number of contact events increases (9.1, 10.9, 12.1, and 13 mN). This could be due an increase in the contact area and/or temporal effects associated with polymer dynamics at the interface.

**Figure 7:**
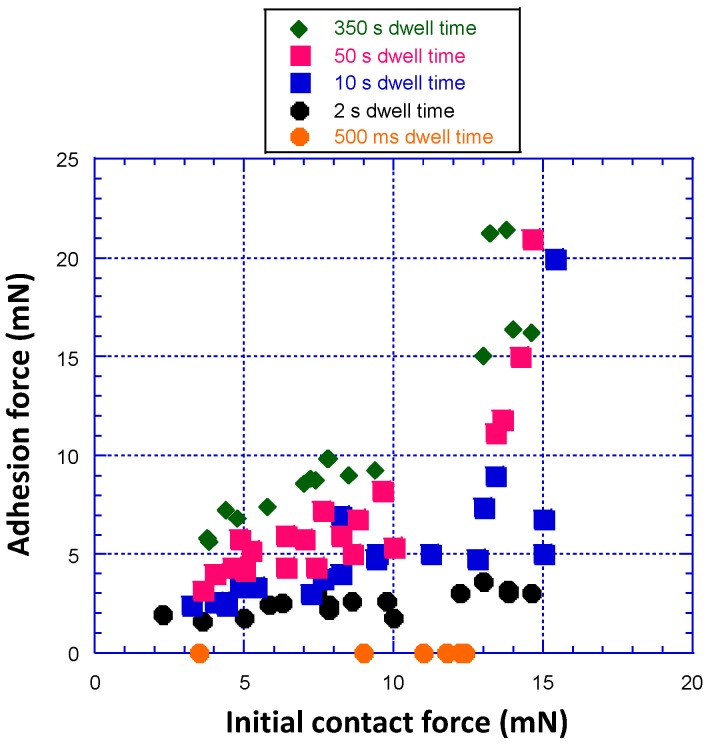
Adhesion force versus initial contact force at various dwell time. Adhesion generally increases with initial contact force and dwell time (notice layering of adhesion force data with respect to dwell time), but no adhesion is observed at the shortest dwell time of 500 ms for any initial contact force. Large adhesion at high dwell time and contact force/area suggest the emergence of an additional adhesion mechanism.

Figure 7 shows adhesion force versus initial contact force at various dwell time. The data were obtained from experiments like the ones shown in Figure 5 and Figure 6. A large number of experiments were conducted so that trends in the data could be identified with confidence. Data are presented in a scatter format to show all of the experimental results. Adhesion generally increases with initial contact force and dwell time, and no adhesion is observed at the shortest dwell time of 500 ms (independent of initial contact force). High initial contact force gives high initial contact area, which increases even further when the material exhibits viscoelastic creep over the dwell time. Thus, both high contact force and long dwell time are associated with high contact area. The τ associated with polymer dynamics at the surface (short segment relaxation plus diffusion/reorientation/ordering) to obtain measurable adhesion is between 500 ms and 2 s based on the data in Figure 7. The time constant for polymer short segment relaxation is relatively short and is applicable to a polymer contact with a hard surface [17]. It is interesting that very large increases in adhesion are observed at higher dwell times (10, 50, and 350 s) for initial contact force equal to or above about 13 mN. Whether this is due to an additional adhesion mechanism that becomes operative at higher contact force and area, or is just a contact area effect requires additional analysis.

**Figure 8:**
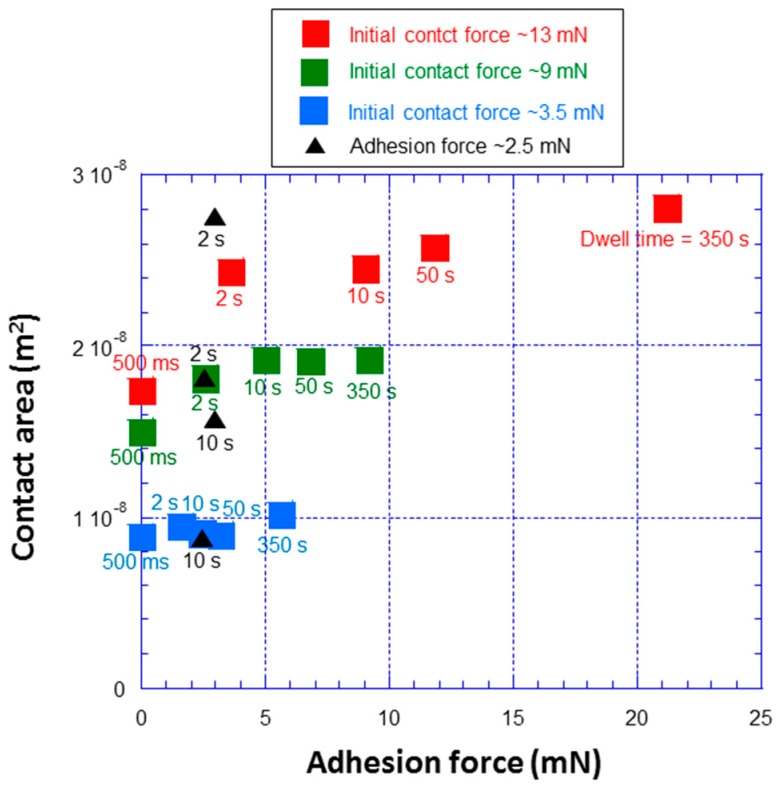
Contact area versus adhesion force at various initial contact force and dwell time. Data are from horizontal and vertical lines in Figure 7. Adhesion increases significantly with dwell time for a given initial contact force. At the highest initial contact force and contact area, adhesion increases even more dramatically at higher dwell times.

Contact area for experiments in Figure 7 can be determined from actuator displacement and force data. It is interesting to see how contact area correlates with adhesion data to try to better understand adhesion mechanisms. Figure 8 shows contact area versus adhesion force at various initial contact force and dwell time. Data are from horizontal and vertical lines in Figure 7. Adhesion increases significantly with dwell time for a given initial contact force. At the highest initial contact force and contact area, adhesion increases even more dramatically with dwell time at higher dwell times. For instance, in going from an initial contact force of 3.5 to 9 mN, the increase in adhesion force (~2 times) at each dwell time is roughly proportional to the increase in contact area (~2 times). This suggests that adhesion force is roughly proportional to contact area. However at the highest initial contact force of 13 mN, the increase in adhesion force at dwell times of 10, 50, and 350 s is much greater than that expected from area considerations alone (area ~2.5 times the 3.5 mN case, but force ~4 times the 3.5 mN case). This behavior suggests that an additional adhesion mechanism is operative at high contact force/area, and that this mechanism has a large time constant compared to the dominant mechanism at low contact area (chain segment relaxation), since it is only operable at high dwell time.

Figure 8 also shows data for adhesion force of about 2.5 mN from Figure 7. Adhesion force of 2.5 mN corresponds to initial contact force and dwell time conditions where polymer chain relaxation is the operative adhesion mechanism. These data are from experiments with dwell times of 2 and 10 s. The 2 s data come from experiments with high initial contact force and high initial contact area, whereas the 10 s data come from experiments with low initial contact force and low initial contact area. The data at ~2.5 mN adhesion force show that adhesion force is not simply determined by contact area. This is a result of polymer chain dynamics Thus, a high contact area at low dwell time gives the same adhesion as a low contact area at long dwell time.

**Figure 9:**
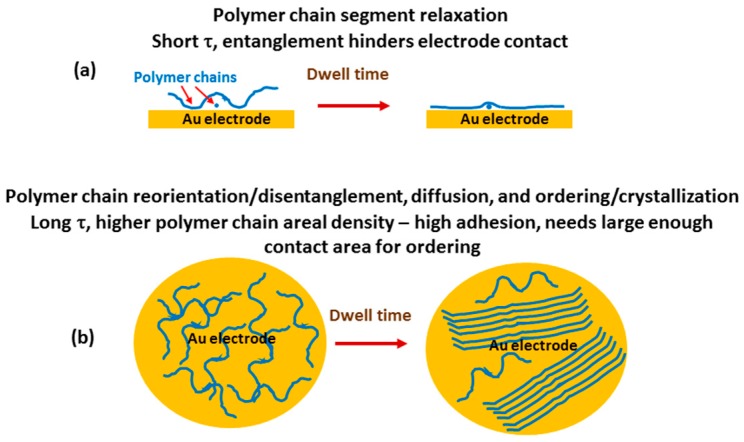
Proposed mechanisms responsible for adhesion behavior in Figure 7 and Figure 8: (**a**) polymer chain relaxation is operative with a short time constant τ and entanglement hinders electrode contact; and (**b**) polymer chain ordering is operative with a long time constant and requires large contact area. This leads to high adhesion due to high packing density of polymer chains.

Figure 9 shows a schematic of the proposed mechanisms responsible for the behavior in Figure 7 and Figure 8. Both mechanisms are driven by energy minimization. Polymer chain segment relaxation into a more intimate contact with the Au electrode with increased dwell time is depicted in Figure 9a. This mechanism has a short τ. It is a relatively fast process because it does not require any displacement or diffusion of the polymer chain to become operative, and only needs the chain to be in close proximity to the electrode surface. Polymer entanglement hinders this mechanism so it is even more relevant in an ordered structure that allows the entire chain to come into intimate contact with the Au electrode.

A more complicated mechanism is illustrated in Figure 9b, which involves polymer chain reorientation/disentanglement, diffusion, and ordering. This mechanism has a longer τ than chain segment relaxation. It leads to a higher polymer chain density in the contact zone and less entanglement, which results in high adhesion due to more polymer chains in contact with the electrode. Adhesion is also increased due to better contact of each chain to the electrode due to less entanglement and complete polymer chain relaxation. This mechanism requires a larger area (contact diameter) for ordering due to extension of the polymer chains with alignment, and is responsible for the sharp increase in adhesion force at high initial contact force and contact area in Figure 7 and Figure 8. A major new contribution to the understanding of adhesion phenomena brought forward in this paper is the concept that polymer chain reorientation/disentanglement, diffusion, and ordering is associated with having enough contact area (contact diameter or dimension larger than the polymer chain length) for the ordered structures to form. This could have major implication for AM, soft microelectromechanical systems (MEMS), and nanoelectromechanical systems (NEMS). Designs should allow for sufficient contact area to provide adequate adhesion between layers to enhance performance and reliability.

**Figure 10:**
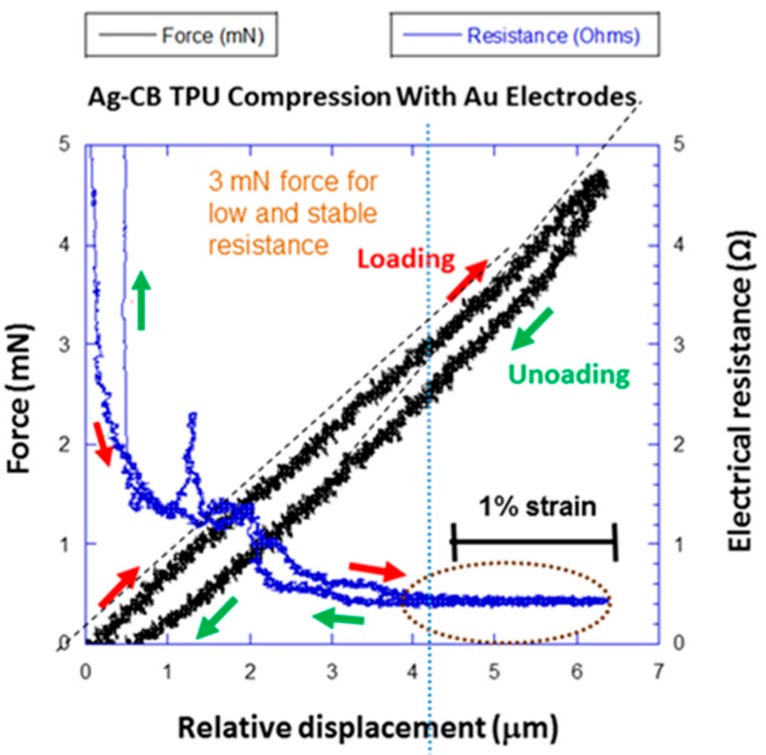
Contact force versus RD for the Ag-CB TPU PNC during a localized compression experiment. The force-RD curve is linear with minimal hysteresis and no adhesion. Resistance is steady and low from 4 to 6.25 μm RD with slightly higher stiffness.

The result of a compression experiment for the Ag-CB TPU PNC is shown in Figure 10. Force is plotted as a function of *RD*. The strain scale bar in Figure 10 is based on the TPU sample thickness of 220 μm. The force-*RD* curve is linear with minimal hysteresis compared to the TPU, and there is no adhesion force. The constant stiffness is due to many spanning conductive paths (high loading of conductive fillers) even at low *RD*/strain, as indicated by measurable low electrical resistance from the onset of the experiment in Figure 10. The spanning conductive paths provide load support through parallel springs [19]. An earlier study with 5 and 10 wt % MWCNT TPU PNCs found that electrical conduction and spanning paths were associated with increased stiffness, but generally did not occur at low *RD*/strain due to the lower conductive filler loadings more representative of sensor applications [19]. There is a slight increase in stiffness above 5 μm *RD* in Figure 10 where resistance is low and steady.

**Figure 11:**
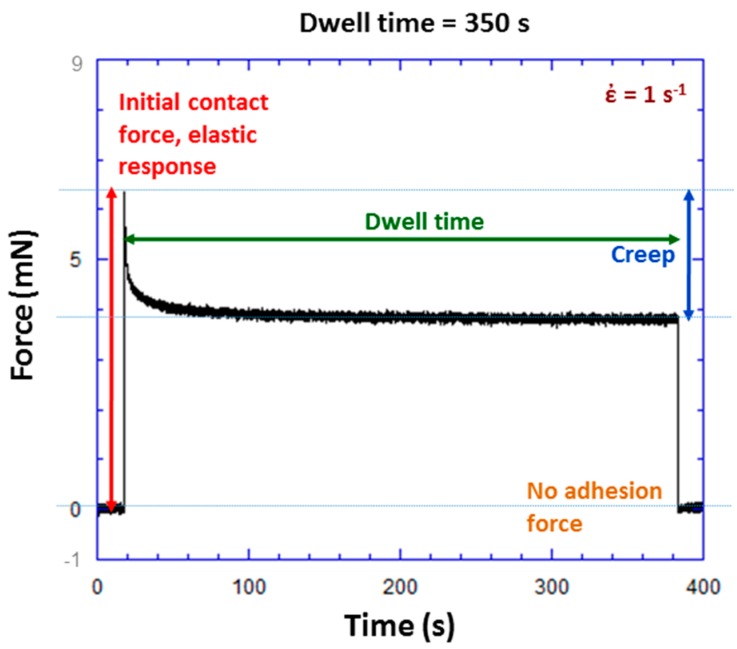
Force versus time during an Ag-CB TPU PNC experiment with a square wave ball excitation. This type of experiment was used to measure adhesion force at various initial contact forces and dwell times. No adhesion force was measured for the PNC.

The lack of adhesion in Figure 10 is due to the rough surface contact between the Au electrodes and the TPU. This limits the RAC and limits the electrode contact with the polymer phase of the TPU and results in no measurable adhesion [19]. Figure 11 shows an example of an adhesion experiment with a square wave voltage excitation (analogous to Figure 5 for the TPU) for the Ag-CB TPU PNC. No adhesion force is seen and was always the case for the PNC over multiple experiments. Although the lack of PNC adhesion may not have major implications for AM design, it could have major implications for MEMS and NEMS switching applications utilizing the PNC and metallic contacts. The lack of adhesion indicates promise for excellent reliability of the switch contacts.

**Figure 12:**
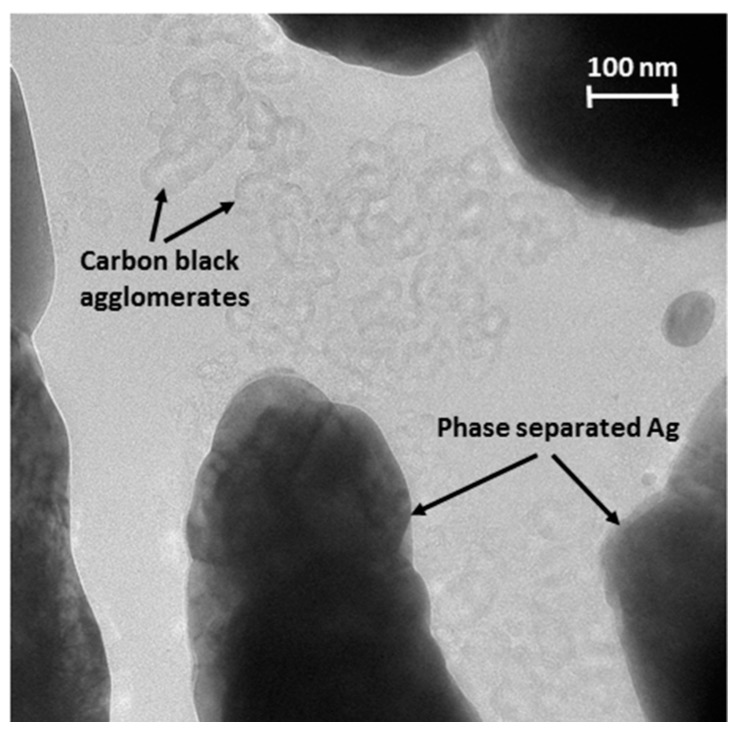
TEM image of PNC showing larger phase separated Ag structures with carbon black agglomerates intervening.

Although subtle, the increase in stiffness coupled with low resistance at high *RD* in Figure 10 is a multiphysics response that provides valuable insight into relevant mechanisms. Figure 12 shows a transmission electron microscope (TEM) image of the PNC. There are larger phase-separated Ag structures with CB agglomerates in between. Under compression, any load bearing spanning paths would initially be a series of Ag and CB agglomerate structures, and high contact pressure on the order of tens of GPa under mN-scale loads will squeeze out any polymer between CB particles along the path [19]. This is due to the very small radii of the CB particles (~15 nm) and the small yield stress of the TPU polymer (tens of MPa). The electrical resistance and stiffness of such a series structure is dominated by the higher resistance and more compliant CB agglomerates. The higher resistance above 1 Ω at low *RD* in Figure 10 is more typical of carbonaceous conduction, whereas the low resistance of about 0.4 Ω at high *RD* is consistent with metallic conduction. Thus, the metallic resistance at high *RD* suggests the emergence of a spanning path(s) comprised of only Ag phase separated structures, which means CB agglomerate structures are squeezed out of some of the conductive paths at high RD. The radius of curvature of the phase-separated silver structures is as low as about 50 nm in Figure 12, which also gives contact pressure on the order of tens of GPa [19]. The mechanical stability of the CB agglomerate bridge structure is not expected to be high, which would allow collapse and squeeze out at high pressure under increasing load/strain. The larger radius of curvature of the metallic structures compared to the CB particles is a likely cause of the subtle increase in stiffness observed at high *RD* in Figure 10.

Minimal hysteresis is observed for the PNC in Figure 10 as compared to the neat TPU in Figure 4. Load support in the PNC is primarily through spanning conductive paths, as opposed to just polymer for the TPU. A spring equivalent model was developed for a MWCNT PNC in a previous study [19]. A spanning path spring in parallel with a more compliant polymer spring is representative of the situation. The stiffness is primarily determined by the spanning path and deflection is relatively small with only a thin layer of the surrounding polymer experiencing the stress field [19]. As such, the expectation for viscoelastic hysteresis is not high in this situation due to only a small cross-sectional area of the polymer being stressed. The present result is consistent with another study where creep resistance of a polyethylene blend was improved with the addition of CB nanoparticles [25].

**Figure 13:**
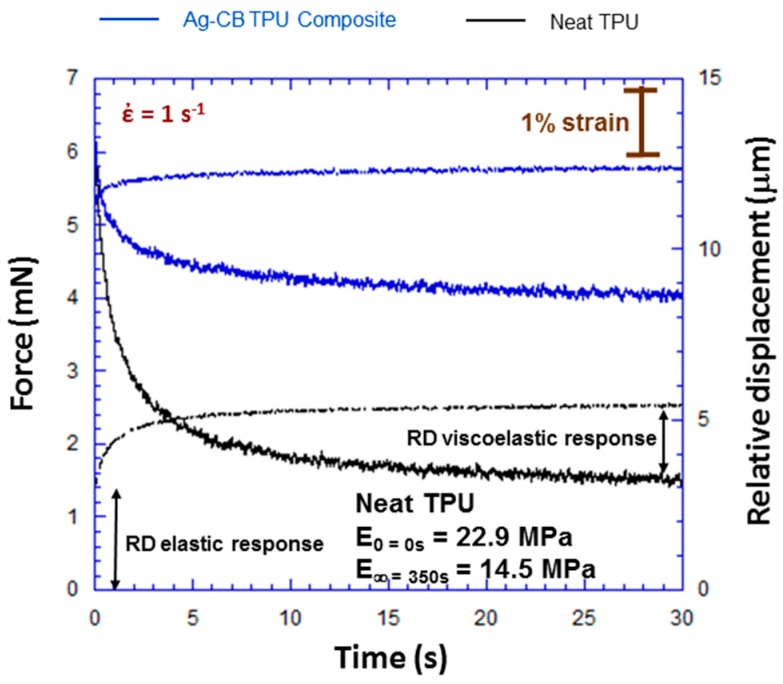
Force and relative displacement versus time during TPU and Ag-CB TPU PNC creep experiments with a square wave ball excitation. This type of experiment was used to measure force relaxation and creep.

Creep experiments were conducted to further characterize the viscoelastic properties of both the TPU and PNC. Figure 13 shows the first 30 of 350 s creep experiments in which a step ball displacement was used to establish contact at a strain rate of 1 s^−1^. For the TPU, the force curve is analogous to the first 30 of the 350 s contact experiment in Figure 5. The elastic response of the *RD* indicates higher stiffness for the neat TPU (less elastic *RD* at *t* = 0). This is a surface roughness and RAC effect and was discussed in detail in an earlier publication [19]. Force relaxation (decrease in force with time) and creep (increase in *RD* due to the viscoelastic response) over time in Figure 13 are larger for the neat TPU as compared to the PNC. This is consistent with the TPU showing more viscoelastic effects than the PNC. Dynamic mechanical analysis (DMA) experiments were conducted and showed a glass transition temperature (*T*_g_) of 3 °C for the PNC with a storage modulus of 1.6 GPa at −90 °C (glassy state) and 550 MPa at 22 °C (experimental temperature). Thus, the viscoelastic behavior shown by the PNC in Figure 13 at 22 °C (above *T*_g_) is consistent with what is expected from the DMA experiments.

The initial and relaxed elastic modulus for the neat TPU can be calculated from the experiment in Figure 13. This is important for viscoelastic characterization of the TPU. Elastic modulus *E* for the TPU was calculated using

*E* = 9*W*/16 *R*^1/2^(*RD*)^3/2^, (4)

where *W* is the load and *R* is the ball radius [26]. Equation (4) is obtained using a Poisson’s ratio of 0.5 for the TPU [27]. The RD is obtained from actuator and wafer displacement data. Although only the first 30 of a 350 s experiment is shown in Figure 13, the omitted data at 350 s were used to calculate the relaxed modulus at 350 s of dwell time. Figure 5 shows a similar full 350 s force relaxation experiment for comparison. The initial elastic modulus was found to be *E*_0 = 0 s_ = 22.9 MPa, and the relaxed modulus at 350 s was found to be *E*_∞ = 350 s_ = 14.5 MPa. This is a 37% decrease in the modulus due to viscoelastic relaxation, and is comparable to another study with PUs that showed viscoelastic behavior with the relaxed modulus at dry-room temperature being 14% (6.5 MPa) lower than the initial modulus (7.4 MPa) [13]. The modulus values found here are within the range of 3.6 to 88.8 MPa that has been reported in the literature for PUs for a room temperature dry environment depending on elastomer composition [13].

It is also important to provide a proper viscoelastic model that fits the experiment. The present experiment presents some difficulties as far as quantitative viscoelastic analysis over the entire experiment. Neither the contact stress nor strain is held constant as is the case for a classic creep or relaxation experiment, respectively. Thus, the viscoelastic characterization provided here is limited as compared to that possible with classic creep or relaxation experiments. In addition, for the PNC, the rough surface and discrete localized asperity contacts (see Figure 2b and Figure 3) create difficulty for calculating the contact stress so the focus of our discussion will be the neat TPU where the average contact stress can be calculated using the contact force and apparent area of contact. Thus, the following discussion of a viscoelastic model and subsequent analysis is for the neat TPU only.

Qualitatively, the viscoelastic model that fits the experiment in Figure 13 is the standard linear model. The standard linear model is Burger’s model minus the Maxwell model dashpot due to the Maxell model viscosity η_M_ being large (η_M_ → ∞) [25]. Burger’s model is the series combination of Maxwell and Kelvin elements [25]. The total strain as a function of time for the standard linear model is

(5) $$\varepsilon_{\mathrm{slm}}=\frac{\sigma }{E_{M}}+\frac{\sigma }{E_{K}}\left(1-e^{\frac{-t}{\tau }}\right),$$

where $\varepsilon_{\mathrm{slm}}$ is the total strain, σ is the contact stress, $E_{M}$ is the elastic modulus of the Maxwell element, $E_{K}$ is the elastic modulus of the Kelvin element, *t* is the time, and τ is the retardation time for the Kelvin unit. The retardation time can be expressed as

(6) $$\tau =\frac{\eta_{K}}{E_{K}},$$

where η_K_ is the Kelvin model viscosity. The shape of the relative displacement curves in Figure 13 is consistent with Equation (5) with the Maxwell elastic displacement at *t* = 0 and the Kelvin exponential increase in relative displacement with time. The recovery data shown in Figure 6 are also consistent with the standard linear model since there is no permanent deformation in the model with the absence of the Maxwell dashpot (full recovery in Figure 6 requires more recovery time and does occur). It is important to note that the viscoelastic behavior may depend on the initial contact stress, so the analysis here should be viewed in the context of an initial contact stress of 0.8 MPa.

The time derivative of Equation (5) yields

(7) $${\dot{\epsilon }}_{\mathrm{slm}}=\;\frac{\sigma }{E_{K}\cdot \tau }\cdot e^{\frac{-t}{\tau }},$$

where ${\dot{\epsilon }}_{\mathrm{slm}}$ is the creep rate of the Kelvin element. Equation (7) can be evaluated at *t* = 0 in conjunction with data in Figure 13. From Figure 13, ${\dot{\epsilon }}_{\mathrm{slm}}$ at *t* = 0 is 1.4 × 10^−2^ s^−1^ and τ is 5 s. Using Equations (6) and (7) at *t* = 0 and a contact stress at *t* = 0 of 0.8 MPa yields $E_{K}$ = 11.8 MPa and η_K_ = 5.9 × 10^7^ Pa·s. The Kelvin element modulus of 11.8 MPa is consistent with the relaxed modulus (*E*_∞=350s_) of 14.5 MPa calculated using Equation (4). The standard linear model is a good fit for the experiment for the contact pressure used in this study.

## 4. Summary/Conclusions

Electromechanical, adhesion, and viscoelastic properties of polymers and polymer nanocomposites (PNCs) are of interest for AM and flexible electronics. Development/optimization of inks for AM is complex, expensive, and substrate/interface dependent. A prescreening, evaluation, and optimization method for ink materials is desirable. This study investigates properties of free standing films of a TPU polymer and an Ag-CB TPU PNC in a lightly loaded low strain compression contact as a rough measure of their suitability for AM. The TPU exhibited high hysteresis and a large viscoelastic response, and sufficient dwell time (500 ms < Δ*t* < 2 s) was needed for polymer chain relaxation and measurable adhesion. A large enough contact area was needed to allow longer time constant polymer ordering in the contact that led to higher adhesion and better performance/reliability. This has implications for interface size relative to polymer chain length in AM design. The standard linear model was found to be a good fit for the viscoelastic behavior of the TPU. The PNC exhibited no adhesion, low electrical resistance, and relatively small viscoelastic response. This implies potential for AM electrical trace as well as MEMS/NEMS switch applications.
